# Heterogenous lung inflammation CT patterns distinguish pneumonia and immune checkpoint inhibitor pneumonitis and complement blood biomarkers in acute myeloid leukemia: proof of concept

**DOI:** 10.3389/fimmu.2023.1249511

**Published:** 2023-09-29

**Authors:** Muhammad Aminu, Naval Daver, Myrna C. B. Godoy, Girish Shroff, Carol Wu, Luis F. Torre-Sada, Alberto Goizueta, Vickie R. Shannon, Saadia A. Faiz, Mehmet Altan, Guillermo Garcia-Manero, Hagop Kantarjian, Farhad Ravandi-Kashani, Tapan Kadia, Marina Konopleva, Courtney DiNardo, Sherry Pierce, Aung Naing, Sang T. Kim, Dimitrios P. Kontoyiannis, Fareed Khawaja, Caroline Chung, Jia Wu, Ajay Sheshadri

**Affiliations:** ^1^Departments of Imaging Physics, University of Texas MD Anderson Cancer Center, Houston, TX, United States; ^2^Departments of Leukemia, University of Texas MD Anderson Cancer Center, Houston, TX, United States; ^3^Departments of Diagnostic Imaging, University of Texas MD Anderson Cancer Center, Houston, TX, United States; ^4^Departments of Pulmonary Medicine, University of Texas MD Anderson Cancer Center, Houston, TX, United States; ^5^Departments of Thoracic/Head and Neck Medical Oncology, University of Texas MD Anderson Cancer Center, Houston, TX, United States; ^6^Departments of Investigational Cancer Therapeutics, University of Texas MD Anderson Cancer Center, Houston, TX, United States; ^7^Departments of Rheumatology and Infectious Diseases, University of Texas MD Anderson Cancer Center, Houston, TX, United States; ^8^Departments of Infectious Diseases, University of Texas MD Anderson Cancer Center, Houston, TX, United States; ^9^Departments of Radiation Oncology, University of Texas MD Anderson Cancer Center, Houston, TX, United States

**Keywords:** habitat analysis, immune checkpoint inhibitor, acute myeloid leukemia, non-small cell lung cancer, pneumonitis

## Abstract

**Background:**

Immune checkpoint inhibitors (ICI) may cause pneumonitis, resulting in potentially fatal lung inflammation. However, distinguishing pneumonitis from pneumonia is time-consuming and challenging. To fill this gap, we build an image-based tool, and further evaluate it clinically alongside relevant blood biomarkers.

**Materials and methods:**

We studied CT images from 97 patients with pneumonia and 29 patients with pneumonitis from acute myeloid leukemia treated with ICIs. We developed a CT-derived signature using a habitat imaging algorithm, whereby infected lungs are segregated into clusters (“habitats”). We validated the model and compared it with a clinical-blood model to determine whether imaging can add diagnostic value.

**Results:**

Habitat imaging revealed intrinsic lung inflammation patterns by identifying 5 distinct subregions, correlating to lung parenchyma, consolidation, heterogenous ground-glass opacity (GGO), and GGO-consolidation transition. Consequently, our proposed habitat model (accuracy of 79%, sensitivity of 48%, and specificity of 88%) outperformed the clinical-blood model (accuracy of 68%, sensitivity of 14%, and specificity of 85%) for classifying pneumonia versus pneumonitis. Integrating imaging and blood achieved the optimal performance (accuracy of 81%, sensitivity of 52% and specificity of 90%). Using this imaging-blood composite model, the post-test probability for detecting pneumonitis increased from 23% to 61%, significantly (*p* = 1.5*E* − 9) higher than the clinical and blood model (post-test probability of 22%).

**Conclusion:**

Habitat imaging represents a step forward in the image-based detection of pneumonia and pneumonitis, which can complement known blood biomarkers. Further work is needed to validate and fine tune this imaging-blood composite model and further improve its sensitivity to detect pneumonitis.

## Introduction

Immune checkpoint inhibitors (ICIs) have been a transformative force in oncology and have become a key part of the therapeutic arsenal for numerous cancers (1). Acute myeloid leukemia (AML), a highly lethal cancer (2) which often requires allogeneic hematopoietic transplantation (allo-HCT) (3) to achieve a durable remission, may sometimes respond to ICIs given in combination with hypomethylating agents (4). However, the use of ICIs to treat AML is associated with high rates of pneumonitis, which significantly increases mortality (5).

A major barrier to diagnosing pneumonitis is the difficulty in distinguishing pneumonitis from other pulmonary conditions, especially pneumonia (6). Bronchoalveolar lavage biomarkers show clonal expensive of Th17.1 cells, but do not necessarily distinguish between pneumonia and pneumonitis (7). Culture-based identification of pathogens can identify up to 60% of infections (8), but these results may require up to 48 hours and are more useful for ruling infection in, and not out. Metagenomic approaches may increase the yield for the detection of bacterial organisms in immunocompromised hosts (9), but the diagnostic yield remains suboptimal for certain infections, and distinguishing colonization from true infection is challenging. The prompt diagnosis of pneumonitis and pneumonia is necessary to ensure the appropriate administration of corticosteroids, both to promptly treat pneumonitis and to be withheld in cases of infection.

Radiomic approaches may allow for the prompt identification of pulmonary disease, as has been shown in interstitial lung diseases (10). However, these approaches have not been tested to distinguish infectious pneumonia from ICI pneumonitis. The classical radiomics approach profiles the infected lung region as a whole entity and may fall short when characterizing phenotypically heterogeneous subareas of the lung that are infected or inflamed. Habitat imaging is an emerging technology that aims to address this challenge by explicitly dividing the region-of-interest (ROI) into coherent subregions termed as habitats (11–13).

Pilot studies from our group and others have demonstrated the added value of habitat imaging analysis in profiling intratumor heterogeneity and predicting treatment response in several cancer types (13–16). In this study, we tested whether our habitat analyses could accurately distinguish pneumonia and pneumonitis in a retrospective cohort of AML patients who received ICIs therapies between 2016-2018 (5).

## Methods

### Participants

We reviewed imaging from a group of 258 patients with AML who were started on ICI therapies (ipilimumab, n=40; nivolumab, n=175; ipilimumab and nivolumab, n=43). between 2016 and 2018. 126 patients with confirmed episodes of pneumonia (n=97) or pneumonitis (n=29) with CT scans available for analysis were included (**Supplementary Figure 1**). All cases were reviewed by a multidisciplinary adjudication committee, who reviewed the clinical history, including the time course of symptoms, representative laboratory, imaging, and microbiological data, and response to antimicrobial or anti-inflammatory therapies (**Table 1**). Pneumonia was diagnosed in episodes with 1) consistent symptoms (e.g. fevers, cough) and consistent imaging (for example, lobar consolidation, nodular opacities, centrilobular or tree-in-bud opacities, cavitary opacities, halo sign) and 2) had a clear response to antibiotics but not corticosteroids or had microbiological confirmation from a lower respiratory tract specimen of an organism known to cause pneumonia (7). Pneumonitis was diagnosed in episodes with 1) consistent symptoms (e.g. cough, shortness of breath) and consistent imaging and 2) a clear response to corticosteroids but not antibiotics or had histopathological confirmation of pneumonitis. Based on CT appearance, pneumonitis cases were classified into the following patterns (17): nonspecific interstitial pneumonitis (NSIP), organizing pneumonia (OP), hypersensitivity pneumonitis (HP), acute interstitial pneumonia (AIP)-acute respiratory distress syndrome (ARDS), or indeterminate/mixed (i.e., nonspecific patchy ground-glass or consolidative opacities or a mixture of patterns without clear dominant pattern). Pneumonitis was graded according to the Common Terminology Criteria for Adverse Events (CTCAE) 5.0 (18). Because symptoms of pneumonitis and pneumonia may often be similar, the multidisciplinary committee weighted imaging, clinical course, and response to therapies heavily in their final diagnoses. The MD Anderson Institutional Review Board approved the study (PA18-0802).

**Table 1:** Characteristics of the study cohort.

Variable	Pneumonia (n=97)	Pneumonitis (n=29)
Median age at enrollment (years)	64.01	69.14
Female sex, n (%)	37(38%)	17(58.6%)
Race, n (%)		
White/Caucasian	84(86.6)	25(86.2%)
Non-white	13(13.4%)	4(13.8%)
AML Diagnosis, n (%)		
*De novo* AML	67(69.1%)	21(72.4%)
Secondary/therapy-related AML	30(30.9%)	8(27.6%)
Prior SCT	17(17.5%)	1(3.4%)
ECOG, n (%)		
0	8(8.25%)	7(24.1%)
1	82(84.54%)	21(72.4%)
2	7(7.21%)	1(3.4%)
Symptoms at baseline, n (%)		
Cough	22(22.7%)	6(20.7%)
Fever	18(18.6%)	7(24.1%)
Shortness of breath	22(22.7%)	8(27.6%)
Symptoms at syndrome, n (%)		
Cough	72(74.2%)	19(65.5%)
Fever	77(79.4%)	21(72.4%)
Shortness of breath	63(64.9%)	22(75.9%)
Median cell counts at baseline		
Bone marrow blasts (%)	20	15
Total WBC (10^3^ cells/μL)	2.5	2.3
NC (cells/μL) %	23	19
LC (cells/μL) %	40	40.1
Platelets (10^3^ cells/μL)	34	29
Median cell counts at syndrome		
Total WBC (10^3^ cells/μL)	2.4	1.4
NC (cells/μL) %	22	30
LC (cells/μL) %	25	25
Platelets (10^3^ cells/μL)	22	14
Smoking status, n (%)		
Never	46(47.4%)	21(72.4%)
Former	48(49.5%)	8(27.6%)
Current	3(3.1%)	
Pneumonia within 30 days of ICI initiation, n (%)	21(21.7%)	6(20.7%)
Viral infection within 30 days of ICI initiation, n (%)	4(4.1%)	1(3.4%)
Prior lung disease, n (%)		
COPD	11(11.3%)	1(3.4%)
Asthma	5(5.2%)	1(3.4%)
Prior autoimmune disease, n (%)	5(5.2%)	2(6.9%)
Chest radiation prior to ICI, n (%)	5(5.2%)	2(6.9%)

ICI, immune checkpoint inhibitor; AML, acute myeloid leukemia; ECOG, Eastern Cooperative Oncology Group; WBC, white blood cell; NC, neutrophil count; LC, lymphocyte count; COPD, chronic obstructive pulmonary disease; ILD, interstitial lung disease; SCT, stem cell transplantation.

### Overall design

**Figure 1:**
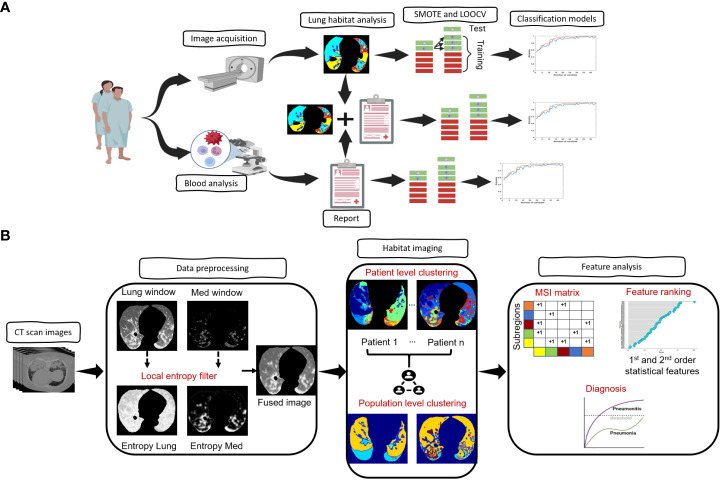
Architecture of the proposed framework. **(A)**, Overview of the overall proposed approach starting from image acquisition, habitat analysis and diagnostic model prediction. **(B)**, Overview of the steps involved in the habitat analysis.

Our overall approach is summarized in **Figure 1A**. In brief, we performed patient and imaging curation, then trained and tested a CT-derived signature using habitat imaging to determine whether a patient was more likely to have pneumonia or pneumonitis. In parallel, we derived a clinical-blood benchmark model by selecting informative clinical and blood metrics to fit into a classification model. Ultimately, we integrated the two approaches (imaging and benchmark features) to evaluate the prediction performance.

### Image acquisition and preprocessing

The CT scans of the 126 patients enrolled in this study were obtained using both Siemens and GE medical systems CT scanners at MD Anderson Cancer Center at the time of the event. CT scans had a slice thickness of 2.5mm and an in-plane spatial resolution of 0.98 to 1.2 mm. A deep learning-based segmentation model (19) was used to extract the left and right lung parenchyma, followed by a morphological dilation and erosion to smoothen the boundaries of the extracted lung regions. An in-house radiologist reviewed and manually corrected the lung ROI segmentations.

### Habitat imaging analysis

The architecture of the habitat imaging technique (16) is a unified approach containing several key steps as illustrated in **Figure 1B**. First, a contrast-enhancing method was applied to filter both the lung and mediastinum window images from the original input images. The extracted lung and mediastinum images were then further processed using a local entropy filter to generate filtered images that capture subtle variations in the texture of the images under different window settings. An image fusion approach was then utilized to combine (fuse) the lung, mediastinum, and their corresponding filtered images to form the final composite image.

Second, the habitat detection has a patient- and population-level clustering blocks. For the patient-level clustering step, the simple linear iterative clustering (SLIC) algorithm (20) is used to oversegment the individual patients’ composite images of lung ROI into a large number of superpixels. Then, the extracted superpixels across the whole patient are aggregated to identify the similar ones inside one patient and across different patients. Specifically, the superpixels from the patient-level clustering step are considered as individual samples. In particular, we characterized individual superpixels by extracting ten features separately on four image channels (CT image normalized by lung window or mediastinal window, as well as two corresponding entropy maps for local texture). Ten different features include skewness, kurtosis, mean, median, 1 quantile, 3 quantile, interquartile range, standard deviation, variance, and energy. These features characterized different aspects of the lung, including the intensity, the symmetricity of intensity and texture, the intensity uniformity, the texture of CT images, and the texture of entropy maps. The superpixels were subsequently clustered using the hierarchical clustering algorithm to identify subregions with similar imaging patterns. The optimal number of subregions was determined using both the gap criterion and hierarchical structure of the clusters dendrogram.

### Feature extraction and machine learning model construction

After the lesion/subregion segmentation, forty-four multiregional spatial interaction (MSI) features were measured from the habitat maps to quantify overall lung parenchyma. In addition, we quantified the symmetric difference ( $\Delta_{Sym}$ ) between left and right lungs by:


(1) $$\Delta_{Sym}\left(\text{L},\text{R}\right)=\left|\text{MS}{\text{I}}_{\text{L}}-\text{MS}{\text{I}}_{\text{W}}\right|\times \left|\text{MS}{\text{I}}_{\text{R}}-\text{MS}{\text{I}}_{\text{W}}\right|$$

where $MSI_{W},\;MSI_{L},\;$ and $MSI_{R}$ denotes the MSI features computed on the whole, left and right lungs, respectively. One strength of the MSI features is their clear interpretations. These features are designed to quantify the spatial heterogeneity of infected lung patterns. Specifically, the MSI features captures information such as the absolute burden and relative percentage of individual habitat as well as their interactions. More detailed explanation regarding the extracted MSI features is presented in **Table 2**.

**Table 2:** Multiregional spatial interaction features interpretation.

Feature name	Feature description
**MSI 1 – MSI 4**	2^nd^ order statistics features (contrast, correlation, Homogeneity and energy)
**MSI 5 – MSI 9**	absolute subregions volume (SR1 – SR5)
**MSI 10 – MSI 14**	interaction (absolute) between subregions and border
**MSI 15 – MSI 18**	interaction (absolute) between SR1 and the remaining subregions, i.e., MSI 15 = SR1 ∩ SR2, MSI 16 = SR1 ∩ SR3, …, MSI 18 = SR1 ∩ SR5.
**MSI 19 – MSI 21**	interaction (absolute) between SR2 and SR3, SR4 and SR5, i.e., MSI 19 = SR2 ∩ SR3, MSI 20 = SR2 ∩ SR4, MSI 21 = SR2 ∩ SR5.
**MSI 22 – MSI 23**	interaction (absolute) between SR3 and SR4, SR5, i.e., MSI 22 = SR3 ∩ SR4, MSI 23 = SR3 ∩ SR5.
**MSI 24**	interaction (absolute) between SR4 and SR5, i.e., MSI 24 = SR4 ∩ SR5.
**MSI 25 – MSI 29**	normalized percentage of subregions volume (SR1 – SR5)
**MSI 30 – MSI 34**	normalized interaction (percentage) between subregions and border
**MSI 35 – MSI 38**	normalized interaction (percentage) between SR1 and the remaining subregions, i.e., MSI 35 = SR1 ∩ SR2, MSI 36 = SR1 ∩ SR3, …, MSI 38 = SR1 ∩ SR5.
**MSI 39 – MSI 41**	normalized interaction (percentage) between SR2 and SR3, SR4 and SR5, i.e., MSI 39 = SR2 ∩ SR3, MSI 40 = SR2 ∩ SR4, MSI 41 = SR2 ∩ SR5.
**MSI 42 – MSI 43**	normalized interaction (percentage) between SR3 and SR4, SR5, i.e., MSI 42 = SR3 ∩ SR4, MSI 43 = SR3 ∩ SR5.
**MSI 44**	normalized interaction (percentage) between SR4 and SR5, i.e., MSI 44 = SR4 ∩ SR5.
**MSI 45 – MSI 48**	symmetric difference (left vs right lung) of the 2^nd^ order statistics features
**MSI 49 – MSI 53**	symmetric difference (left vs right lung) of absolute subregions volume (SR1 – SR5)
**MSI 54 – MSI 58**	symmetric difference (left vs right lung) of the interaction (absolute) between tumor subregions and border
**MSI 59 – MSI 62**	symmetric difference (left vs right lung) of the interaction (absolute) between SR1 and the remaining subregions, i.e., MSI 59 = |*MSI_L_* 15 – *MSI_W_* 15| × |*MSI_R_* 15 – *MSI_W_* 15|, …, MSI 62 = |*MSI_L_* 18 – *MSI_W_* 18| × |*MSI_R_* 18 – *MSI_W_* 18|.
**MSI 63 – MSI 65**	symmetric difference (left vs right lung) of the interaction (absolute) between SR2 and SR3, SR4 and SR5, i.e., MSI 63 = |*MSI_L_* 19 – *MSI_W_* 19| × |*MSI_R_* 19 – *MSI_W_* 19|, …, MSI 65 = |*MSI_L_* 21 – *MSI_W_* 21| × |*MSI_R_* 21 – *MSI_W_* 21|.
**MSI 66 – MSI 67**	symmetric difference (left vs right lung) of the interaction (absolute) between SR3 and SR4, SR5, i.e., MSI 66 = |*MSI_L_* 22 – *MSI_W_* 22| × |*MSI_R_* 22 – *MSI_W_* 22|, MSI 67 = |*MSI_L_* 23 – *MSI_W_* 23| × |*MSI_R_* 23 – *MSI_W_* 23|.
**MSI 68**	symmetric difference (left vs right lung) of the interaction (absolute) between SR4 and SR5, i.e., MSI 68 = |*MSI_L_* 24 – *MSI_W_* 24| × |*MSI_R_* 24 – *MSI_W_* 24|
**MSI 69 – MSI 73**	symmetric difference (left vs right lung) of the percentage of subregions volume (SR1 – SR5)
**MSI 74 – MSI 78**	symmetric difference (left vs right lung) of the normalized interaction (percentage) between subregions and border
**MSI 79 – MSI 82**	symmetric difference (left vs right lung) of the normalized interaction (percentage) between SR1 and the remaining subregions, i.e., MSI 79 = |*MSI_L_* 35 – *MSI_W_* 35| × |*MSI_R_* 35 – *MSI_W_* 35|, MSI 82 = |*MSI_L_* 38 – *MSI_W_* 38| × |*MSI_R_* 38 – *MSI_W_* 38|.
**MSI 83 – MSI 85**	symmetric difference (left vs right lung) of the normalized interaction (percentage) between SR2 and SR3, SR4 and SR5, i.e., MSI 83 = |*MSI_L_* 39 – *MSI_W_* 39| × |*MSI_R_* 39 – *MSI_W_* 39|, MSI 85 = |*MSI_L_* 41 – *MSI_W_* 41| × |*MSI_R_* 41 – *MSI_W_* 41|.
**MSI 86 – MSI 87**	symmetric difference (left vs right lung) of the normalized interaction (percentage) between SR3 and SR4, SR5, i.e., MSI 86 = |*MSI_L_* 42 – *MSI_W_* 42| × |*MSI_R_* 42 – *MSI_W_* 42|, MSI 87 = |*MSI_L_* 43 – *MSI_W_* 43| × |*MSI_R_* 43 – *MSI_W_* 43|.
**MSI 88**	symmetric difference (left vs right lung) of normalized interaction (percentage) between SR4 and SR5, i.e., MSI 88 = |*MSI_L_* 44 – *MSI_W_* 44| × |*MSI_R_* 44 – *MSI_W_* 44|.

MSI_L_, multi regional spatial interaction feature extracted from the left lung; MSI_R_, multi regional spatial interaction feature extracted from the right lung; MSI_W_, multi regional spatial interaction feature extracted from the whole lung.

After feature extraction, the correlation among the extracted features was explored. Also, the univariate Chi-square test statistics approach was applied to examine feature association with infection types. Each feature was tested independently, and the output of the univariate Chi-square model is the probability (*p*-value) that the patient had been diagnosed with either pneumonia or pneumonitis for each feature. The computed *p*-values of all the features are then used to rank the individual features by computing feature importance (score) as:


score= −log(p)

where *p* is the corresponding *p*-value for each feature, and higher score denotes greater importance. Next, we iteratively increase the number of selected features based on their importance in order to identify the optimal diagnostic model. Specifically, the top-ranked features were used to build an ensemble model of 100 boosted classification trees. To avoid biased classification to the pneumonitis class due to data imbalance (i.e., significantly larger number of samples in the pneumonia class), we employed synthetic minority oversampling technique (SMOTE) nested with leave-one-out cross validation (LOOCV) approach together to validate model performance. To avoid information leakage, we first left out one sample as the test set before applying SMOTE on the training set to train a prediction model. This process is repeated *n* -times (*n* equals to total number of samples) until every data sample is left out as a test sample.

In parallel, we also built a benchmark model using clinical and blood-based measures. For clinical variables, we included cough, fever, shortness of breath at both baseline and at time of syndrome together with age and sex. For blood-based variables, we used five blood-based measurements including absolute white blood cells (WBC) count, absolute neutrophils count (ANC), absolute lymphocyte count (ALC) and platelet count at both baseline and time of syndrome together with bone marrow blast cells count at baseline. Using similar strategy as our earlier work (5), we considered the log transformation of WBC and platelets at both baseline and at time of syndrome. Given the clinical and blood measures, we adopted a similar SMOTE and LOOCV machine learning strategy to build the benchmark model. The diagnostic performance of the benchmark model was compared to the habitat imaging model. Furthermore, we evaluated the performance when integrating clinical-blood benchmark and habitat in a composite model. For comparison purposes, we also extracted the classical radiomics features from the whole lung regions and built a prediction model.

### Statistical analysis

The ability to separate pneumonia (coded as 0) from pneumonitis (coded as 1) was assessed by the accuracy, specificity, and sensitivity in the leave-one-out cross-validation. For this work, sensitivity means the true positive rate of pneumonitis, while specificity represents true negative rate of pneumonitis. To mitigate the imbalance in the distribution of pneumonia and pneumonitis, Synthetic Minority Oversampling Technique (SMOTE) algorithm was applied. Furthermore, we applied Bayesian theorem to compute the pre-test and post-test probability (21), which referred to the probability of detecting pneumonitis before a diagnostic model was performed (pre-test probability) and after a model is performed (post-test probability). The feature correlation analysis was done using the Pearson’s correlation test with the R software. The Chi-square test statistics was used to evaluate the predictive value of individual features and the t-test statistics was used to compare the prediction performance of the different models.

## Results

### Study participants

**Table 1** shows the characteristics of the overall study cohort (n=126) who developed pneumonia or pneumonitis. Most patients had *de novo* AML, but ICI was usually given after frontline therapy was initiated. 86 patients received nivolumab without ipilimumab, either alone (n=11), or with azacitidine (n=58) or idarubicin (n=17). 15 patients received ipilimumab without nivolumab, either alone (n=9) or with azacitidine (n=6). 25 patients received nivolumab and ipilimumab together, with (n = 17) or without (n=8) azacitidine. We identified 97 distinct patients of pneumonia and 29 distinct patients of pneumonitis in which a CT was available for analysis. No patients had more than one pneumonia or pneumonitis. All cases of pneumonia and pneumonitis were independently reviewed by blinded expert thoracic radiologists who reviewed feature characteristics in the current study. Representative cases of pneumonia and pneumonitis are shown in **Supplementary Figure 2**. **Supplementary Table 1** shows a list of organisms isolated in cases of microbiologically-proven pneumonia. Of the patients with pneumonitis, 19 had an indeterminate/mixed pattern, 7 had an organizing pneumonia pattern, and 3 had an acute interstitial pneumonia (AIP)-acute respiratory distress syndrome (ARDS) pattern. The median time to pneumonitis was 109 days after ICI initiation (range 1-484 days).

### Habitat imaging reveals intrinsic infection patterns of lung parenchyma

**Figure 2:**
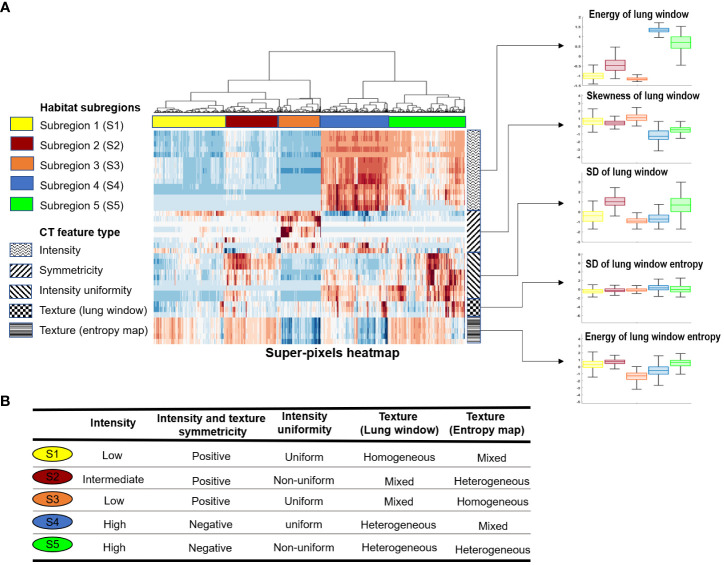
Habitat subregions identification. **(A)**, Heatmap of the five identified subregions together with distribution plot (boxplot) of five representative features from the feature types grouped by habitat subregions. **(B)**, Characteristics of the five habitat subregions in relation to the CT feature types.

**Figure 3:**
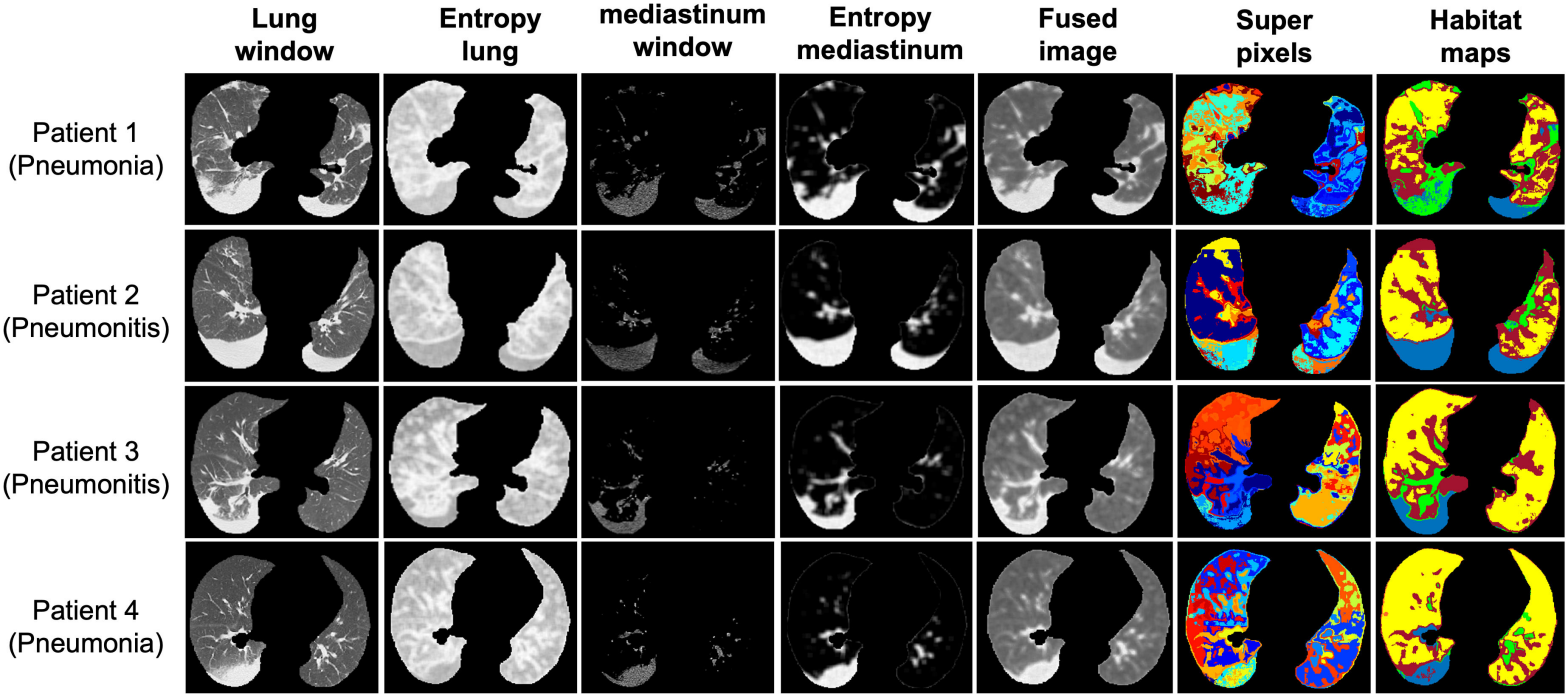
Representative habitat maps of four subjects from both the pneumonia and pneumonitis categories.

We applied our proposed habitat imaging method (**Figure 1B**) and determined the optimal number of intra-lung subregions. As shown in **Figure 2A**, there are five distinct clusters (i.e, habitats) according to hierarchical structure of the dendrogram. We then investigated the imaging parameters that underline and differentiate these habitats. **Figure 2A** shows the detailed distributions of the representative features from five types of imaging parameters (intensity uniformity, Intensity, texture of lung window, texture of entropy map, symmetricity of intensity and texture) in each of these habitats. The detailed phenotypical patterns of CT imaging associated with each habitat were summarized in **Figure 2B**. We observed that subregions 1 corresponds to the uninfected lung parenchyma, subregion 3 corresponds to ground glass opacity (GGO) with elevated texture heterogeneity and low CT number, subregion 4 corresponds to the consolidation, subregions 2 and 5 correspond to the transition zone to GGO at different degrees. The detailed partitioning results of entire lung region after habitat analysis were presented in **Figure 3** for four selected pneumonia and pneumonitis patients, where detailed habitats were consistently defined to quantify the infection patterns.

### Habitat model outperforms benchmark model of clinical and blood metrics

**Figure 4:**
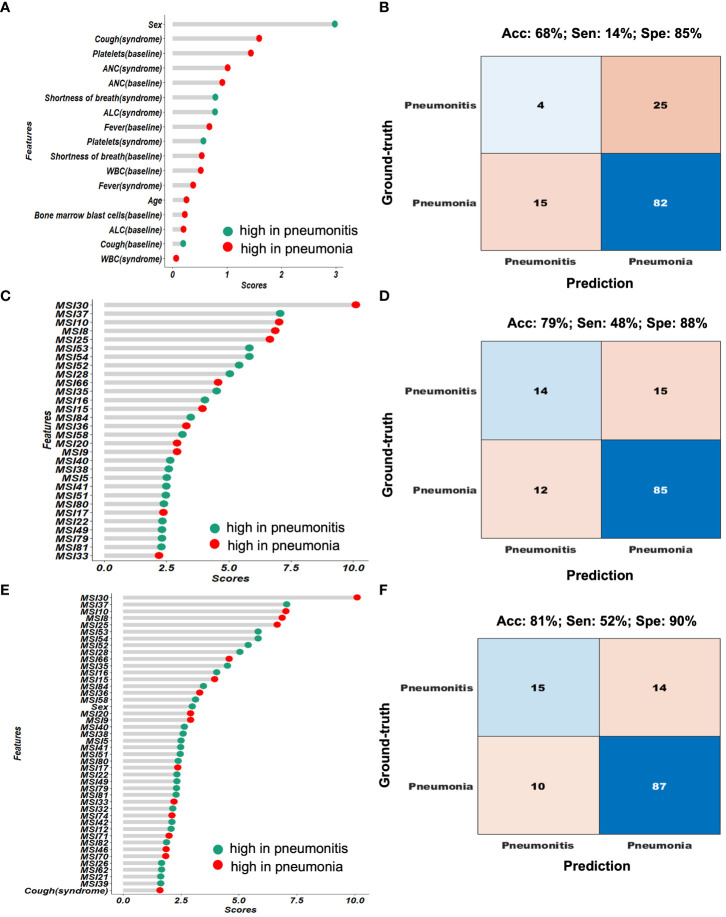
Performance comparison of the different diagnostic models. **(A, B)** shows the feature importance and confusion matrix for the clinical-blood (benchmark model). **(C, D)** shows the feature importance and confusion matrix for the habitat-based model. **(E, F)** shows the feature importance and confusion matrix for the composite (clinical-blood plus habitat models).

Given the clinical variables and blood metrics both at baseline and at time of infection (their correlation in **Supplementary Figure 3A**), we built a benchmark model with the feature importance presented in **Figure 4A** and model performance in **Figure 4B**. The top ranked features include sex; cough at time of event; baseline platelets; and ANC (at both baseline and at time of event). This benchmark model had an accuracy of 68%, sensitivity (i.e., true positive of pneumonitis) of 14%, and specificity (true negative of pneumonitis) of 85%. Then, based on the habitat map for individual patients, we extracted MSI features to characterize the overall infection patterns as well as their symmetricity between left and right lungs, which resulted in a total of 88 features. The correlation among these habitat features was presented in **Supplementary Figures 3B, C**. Next, we built a classifier to differentiate pneumonia from pneumonitis using LOOCV, and the feature importance was presented in **Figure 4C**. For the prediction model, interestingly the top ranked habitat features were MSI30 which measures the infected area on the lung surface and MSI37, which relates to the interaction between habitat 1 (normal lung parenchyma) and habitat 4 (consolidation). Using this approach, we found that pneumonia had elevated asymmetric interaction, suggesting more asymmetry in the CT pattern between left and right lung. The habitat model achieved an accuracy of 79%, sensitivity of 48%, and specificity of 88%, based on the cross-validated confusion matrix in **Figure 4D**. For comparison purposes, we also built a conventional radiomics model (**Supplementary Figure 4**), which we found to have a significantly lower performance (accuracy, 60%; sensitivity, 17%; specificity, 72%) to the habitat model (*p* = 5*E* − 19).

### Imaging-blood composite model achieves the optimal performance

**Figure 5:**
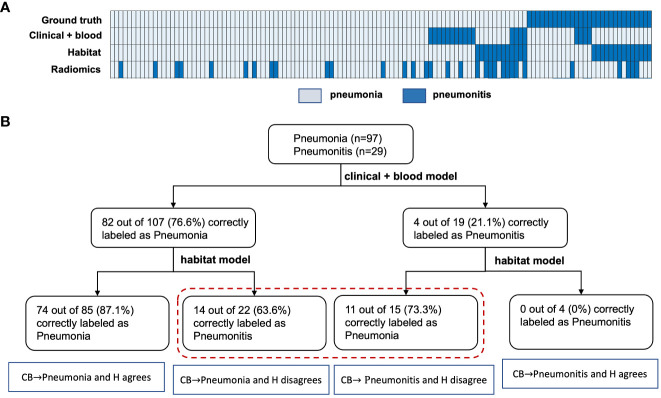
Evaluation of the benefit of the habitat model in improving baseline model prediction. **(A)**, shows the heatmap of the different model’s prediction. **(B)**, shows how the cascading model improves the benchmark models predictions in both the pneumonia and pneumonitis group. Notes that CB and H here represents the clinical-blood and habitat models, respectively.

Next, we integrated the prediction results from both the habitat model and the benchmark model (**Figure 5A**). Based on the cascading model with clinical and blood model in the first layer and habitat model in the second, we simulated the predicted infection type stratification as shown in **Figure 5B**. If we set up the rule as following: we will make a diagnosis if both models agree and will label the cases as ambiguous cases if both models disagree. This has achieved 87.1% accuracy in predicting pneumonia, a greater than 10% increase than clinical model. Of note, 0% accuracy in predicting pneumonitis, indicating imaging and blood are capturing different and non-overlapping pneumonitis cases. For these 37 conflicting cases that imaging and blood model disagreed, imaging model had an accuracy of 59% and the blood model had an accuracy of 41%.

In addition, we mixed the habitat features with clinical and blood metrics together to re-fit a prediction model, and the ranked feature importance table was shown in **Figure 4E**. In general, the habitat features were consistently more important than the clinical and blood measures. The composite model achieved the optimal performance with an overall accuracy of 81%, sensitivity of 52% and specificity of 90% (**Figure 4F**).

Further, we compared the post-test probability of detecting pneumonitis based on different models when the various models diagnosed pneumonitis (**Table 3**). The pre-test probability (i.e., prevalence) of pneumonitis was observed to be 23%. With the benchmark prediction model using clinical and blood metrics, the post-test probability degraded to 22% if the model diagnosed pneumonitis. When using our habitat model, the post-test probability increased to 55% if the model diagnosed pneumonitis. We observed synergistic effects when combining habitat imaging with clinical and blood metrics into a composite model, which achieved the best post-test probability of 61% if the model diagnosed pneumonitis, more than a 2-fold increase from pre-test probability. By contrast, the classical radiomics model had the worst performance with post-test probability of 15%.

**Table 3:** Pretest and posttest probability comparison among different diagnostic models.

Model	Pre-test Probability	Post-test Probability
Benchmark (clinical-blood)	23%	22%
Habitat		55%
Refitted composite model		61%
Classical Radiomics		15%

## Discussion

Recognizing the difficulty in distinguishing pneumonia and pneumonitis in the absence of definitive biomarkers, we developed an imaging-based pipeline that could distinguish these two entities with a much-improved accuracy using a well-characterized cohort of patients with AML undergoing ICI therapy. Our proposed imaging marker has significantly outperformed a benchmark model based on clinical-blood metrics. Further, we observed a synergy between our imaging markers and blood markers, and their integration into a joint model has achieved the best prediction. All in all, our pilot study serves as proof-of-concept to demonstrate that machine learning of computed tomography (CT) scans can offer complementary values on top of existing clinical biomarkers for improved management of immune-related adverse events (irAE).

Diagnosing pneumonitis in real-time is challenging, and the prompt differentiation of pneumonitis from other conditions, such as pneumonia or cancer progression, is not always possible from imaging information alone. Additional tests may be required, but these results may further delay the prompt administration of definitive therapy toward pneumonia or pneumonitis, potentially leading to patient harm. For example, prompt administration of antibiotics for community acquired pneumonia decreases in-hospital mortality (22). Improving the ability of interpreting radiologists to diagnose pneumonitis may lead to improved patient outcomes. Tools such as the one we highlight in this work can potentially augment the capabilities of interpreting so to help radiologists make definitive image-based diagnoses. Approaches that combine artificial intelligence imaging tools with clinical radiologists often exceed the accuracy seen with human evaluations, as has been shown when determining the probability that a lung nodule is malignant (23) or whether reticular abnormalities represent interstitial lung disease (24). We envision that this tool may reduce the uncertainty seen when trying to differentiate pneumonia and pneumonitis in real time, but further studies are needed to validate this.

Pneumonitis is a serious complication of checkpoint inhibitor immunotherapy, and the mortality ranges from 10-20% in non-small cell lung cancer cohorts (NSCLC), where ICIs are frequently used (25–27) to nearly 50% in AML (5). Pneumonitis is likely to be more amenable to treatment if detected early and distinguished from pneumonia. The treatments for pneumonitis, namely immunosuppressive therapies of appropriate intensity and duration, are substantively different from the treatments for pneumonia. Furthermore, overuse of antimicrobial agents in patients without pneumonia may alter the intestinal microbiome, potentially reducing the efficacy of ICIs (28). In our original report, 28/31 cases of pneumonitis were treated with both corticosteroids and antibiotic therapies. While pneumonia was fivefold more common in our cohort than pneumonitis, promptly distinguishing these two conditions will benefit all patients undergoing ICI therapy for cancer, regardless of the underlying rates of these two conditions. Further, diagnosis of pneumonitis with histopathology in nearly all cases due to the concern for bleeding due to thrombocytopenia or the concern for pulmonary deterioration after a biopsy procedure.

Computed tomography (CT) patterns associated with immune checkpoint inhibitor related pneumonitis may resemble interstitial lung diseases seen in the general population, including organizing pneumonia, interstitial pneumonitis, and others (29). The patterns that may be seen in these diseases is highly variable from case to case, as others have shown (30, 31). Radiomics has been used to predict the risk of developing ICI-induced pneumonitis based on baseline CT scans from 2 patients who developed pneumonitis and 30 who did not (32), but not to differentiate pneumonitis from other lung diseases. In this study, we have implemented the habitat imaging algorithm to differentiate pneumonia and pneumonitis. Compared to conventional radiomics, the key strength of our habitat imaging analysis is that it explicitly accounts for the spatial heterogeneity of the infected lung and partitions the whole lung regions into phenotypically distinct subregions. By analyzing these subregions individually as well as their interactions, we have demonstrated its superior performance in separating pneumonitis from pneumonia. Analogous to the superior multiregional gene sequencing over conventional cocktail sequencing (33), a fine grained spatial analysis enabled by habitat imaging can reveal new insights to improve the pneumonitis diagnosis. By contrast, traditional radiomics extracts features (including texture) from the entire lung region but cannot capture the degree of intra-lung infection heterogeneity. This may explain why our habitat imaging approach outperformed conventional radiomics.

Our study has several strengths. This is the first tool of its kind and is positioned to address a significant problem that hinders the treatment of all patients undergoing ICI therapy. The tool was developed by incorporating CT images that used diverse acquisition protocols, and thus can be more easily applied and validated in external cohorts. Also, another strength of our study is the strict selection of patients with AML under immunotherapy. AML patients do not have solid malignancies in their lung regions to confound the imaging analysis, which is different from solid tumors (e.g. NSCLC).

Several limitations must be considered. First, the results presented in this manuscript would benefit from an external cohort for model validation. Second, while all cases of pneumonia and pneumonitis in this study were confirmed by an expert multidisciplinary cohort at MD Anderson, the accuracy of this tool needs to be confirmed in a prospective cohort where the appropriate testing, especially CT imaging of the chest and universal BAL, are performed promptly and systematically. Third, it is likely that blood and clinical markers that associate with pneumonitis will vary from cohort to cohort, which would make an approach that only utilizes imaging more attractive. Fourth, other lung processes such as disease progression or radiation injury are more applicable to solid tumors but not seen in the current cohort treated for AML, and therefore this tool must be validated before applying in patients with solid tumors such as non-small cell lung cancer. Fifth, it is possible that pneumonia and pneumonitis may co-exist in some patients, and it is not uncommon for more than one serious adverse event to manifest concurrently in AML patients (34). Therefore, a test that determines the probability of one or the other as mutually exclusive results may not be appropriate in all cases. Sixth, there is no “gold standard” to diagnose pneumonia, and it remains a clinical diagnosis. Therefore, it is possible that the multidisciplinary adjudication of pneumonia and pneumonitis were erroneous in some instances. Finally, because ICIs are not currently approved to treat AML, there is not a possibility to expand the number of cases with pneumonitis in a similar cohort.In conclusion, we developed a tool that could accurately distinguish pneumonia and pneumonitis in AML patients treated with ICI inhibitors. If validated, our approach holds great promise to improve the clinical care of cancer patients treated with ICIs by improving our ability to differentiate pneumonitis from other lung diseases in a prompt fashion.

## Data availability statement

The data of this study are available through signed data access agreement from the corresponding author. De-identified blood and clinical data will be provided on reasonable request. The CT image data are not publicly available since they contain sensitive information that could compromise patient privacy.

## Ethics statement

The studies involving humans were approved by MD Anderson Institutional Review Board. The studies were conducted in accordance with the local legislation and institutional requirements. The ethics committee/institutional review board waived the requirement of written informed consent for participation from the participants or the participants’ legal guardians/next of kin because Waiver of consent for this study. Original study consented patients for the trial. Written informed consent was not obtained from the individual(s) for the publication of any potentially identifiable images or data included in this article because Waiver of consent for this study. Original study consented patients for the trial.

## Author contributions

AS and JW conceived the study. All authors participated in data collection. MuA performed primary data analyses with the guidance of AS and JW. MuA, JW, and AS wrote the manuscript. All authors contributed to the article and approved the submitted version.
