# Supplementary material for: Heterogenous lung inflammation CT patterns distinguish pneumonia and immune checkpoint inhibitor pneumonitis and complement blood biomarkers in acute myeloid leukemia: proof of concept
Source: Front Immunol. 2023 Sep 29;14:1249511. doi: 10.3389/fimmu.2023.1249511 (PMC10570510; doi:10.3389/fimmu.2023.1249511)
Supplement: Supplementary file 1 [file DataSheet_1.docx]

Supplementary Material

Heterogenous lung inflammation CT patterns distinguish pneumonia and immune checkpoint inhibitor pneumonitis and complement blood biomarkers in acute myeloid leukemia: proof of concept

Muhammad Aminu^1^, Naval Daver^2^, Myrna C.B. Godoy^3^, Girish Shroff^3^, Carol Wu^3^, Luis F. Torre-Sada^4^, Alberto Goizueta^4^, Vickie R. Shannon^4^, Saadia A. Faiz^4^, Mehmet Altan^5^, Guillermo Garcia-Manero^2^, Hagop Kantarjian^2^, Farhad Ravandi-Kashani^2^, Tapan Kadia^2^, Marina Konopleva^2^, Courtney DiNardo^2^, Sherry Pierce^2^, Aung Naing^6^, Sang T. Kim^7^, Dimitrios P. Kontoyiannis^8^, Fareed Khawaja^8^, Caroline Chung^9^, Jia Wu*^1^, Ajay Sheshadri*^4^

*** Co-senior authors**

Corresponding Author: [jwu11@mdanderson.org](mailto:jwu11@mdanderson.org)

**
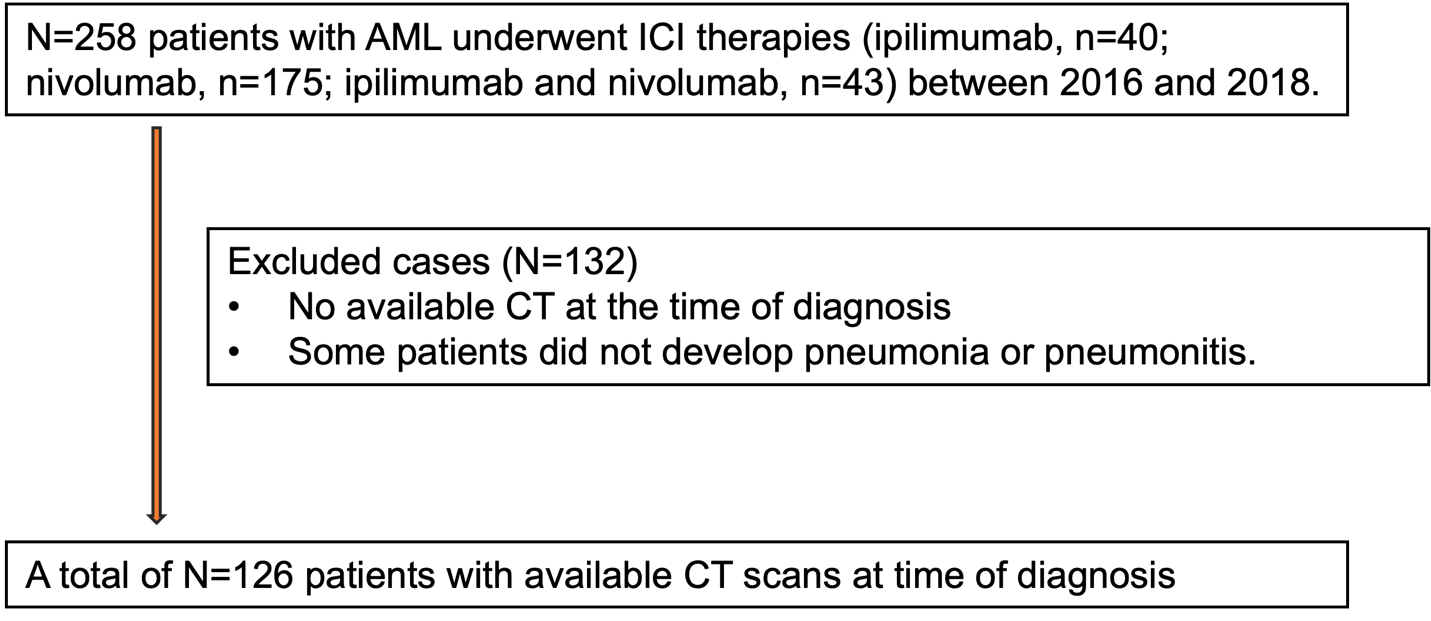
**

**Supplementary Figure 1.** Flowchart depicting the inclusion and exclusion criteria for the patients considered in the analysis.

**
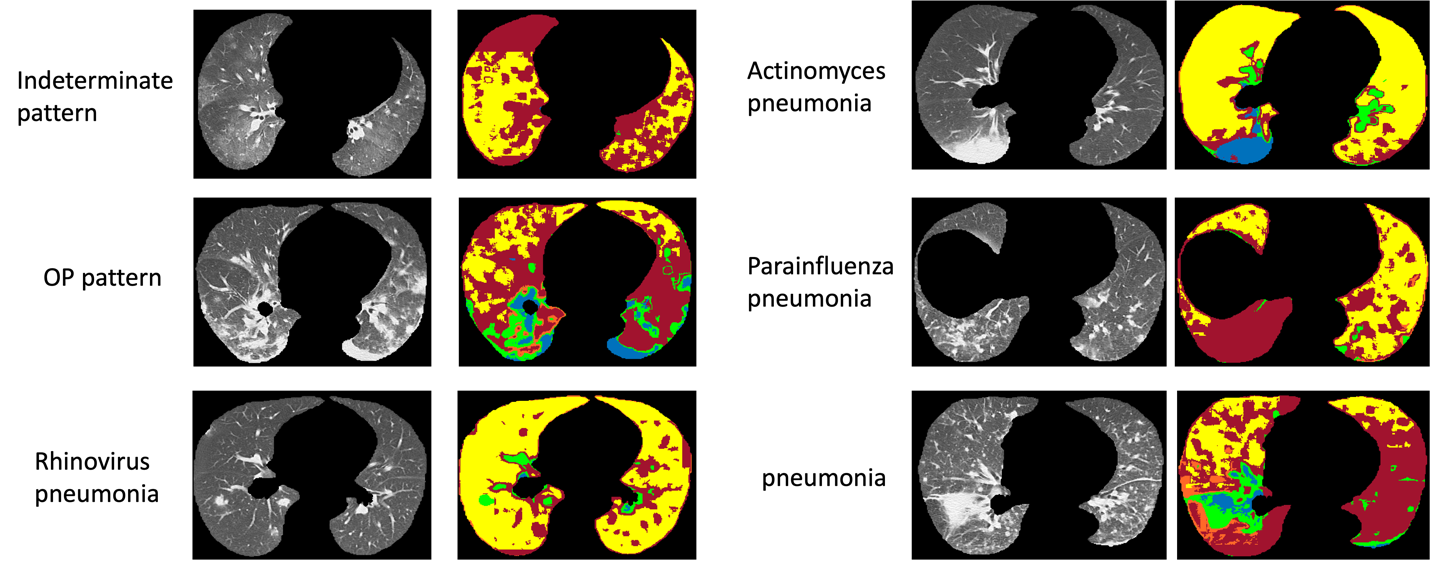
**

**Supplementary Figure 2.** Example of different types of Pneumonia with corresponding habitat maps.

**
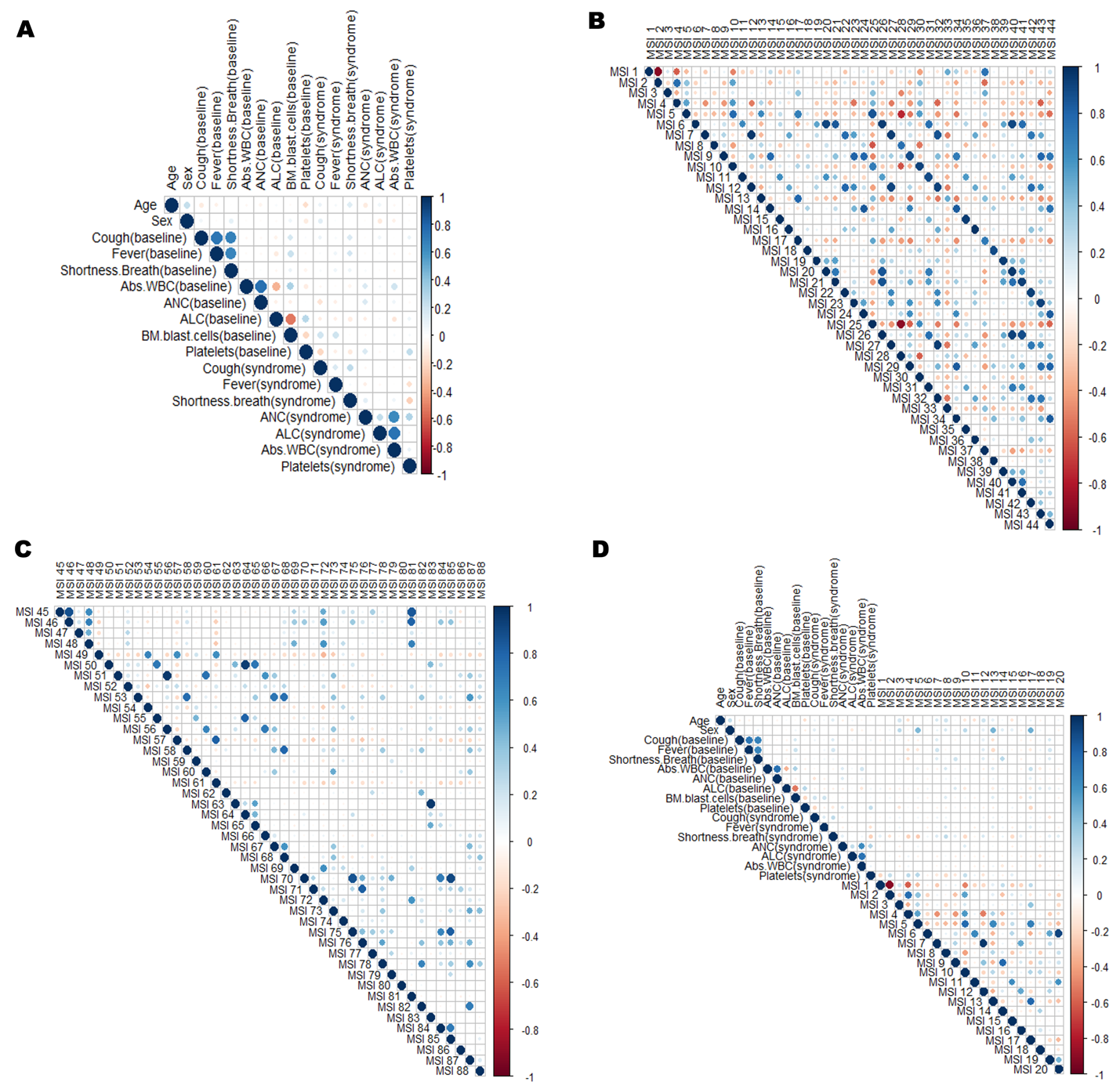
Supplementary Figure 3.** Correlation heatmaps for features used to build the different diagnostics models. A – D, Shows the correlation heatmaps for the clinical-blood features, MSI features extracted from the whole lung, MSI features measuring the symmetricity between the left and right lung, and combination of the whole features. Note, in D, we only show the correlation among the clinical-blood and MSI 1 – MSI 20 due to the large number of features.

**
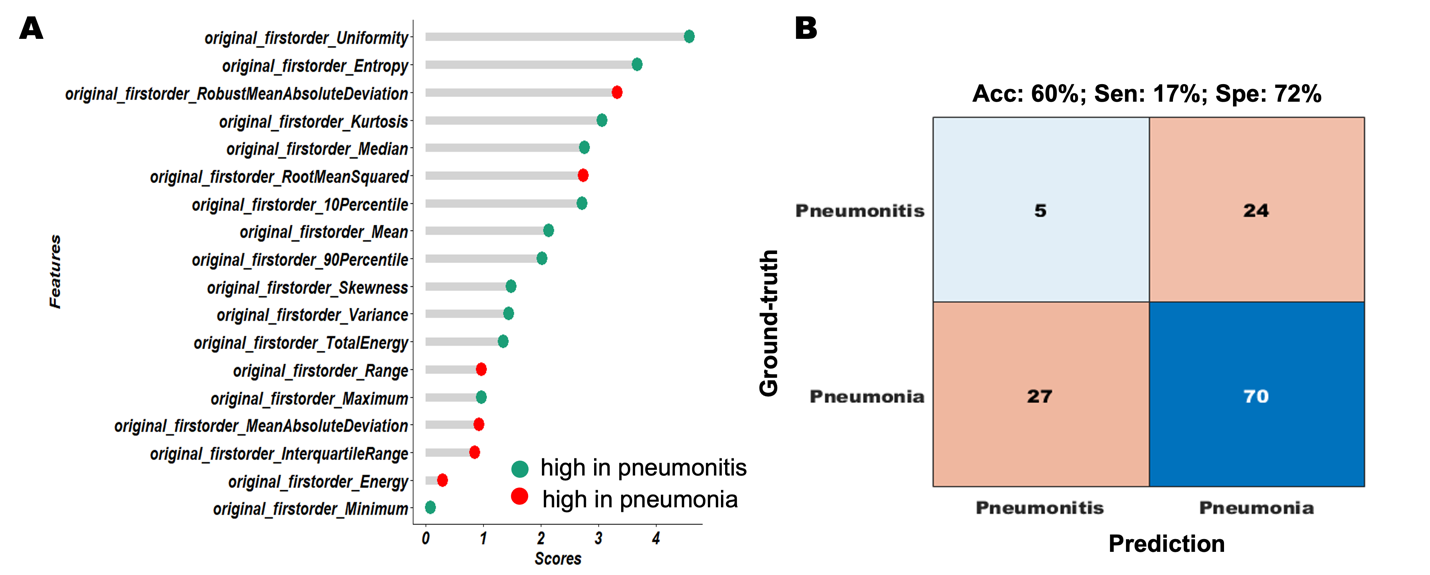
Supplementary Figure 4.** Evaluation of the radiomics diagnostic model. A and B shows the feature importance and the model’s performance in terms of confusion matrix.

**Supplementary Table 1. Summary of microbiological results for 97 cases with pneumonia***

| Type of infection | Species | n |
| --- | --- | --- |
| Bacterial (n=29) | Methicillin-resistant Staphylococcus aureus | 6 |
|  | Coagulase-negatiev Staphylococcus spp | 5 |
|  | Stenotrophomonas maltophilia | 4 |
|  | Pseudomonas aeroginosa | 3 |
|  | Klebsiella pneumoniae | 3 |
|  | Enterococcus faecalis | 2 |
|  | Actinomyces odontolyticus | 2 |
|  | Streptococcus pneumoniae | 1 |
|  | Haemophilus parainfluenzae | 1 |
|  | Legionella pneumophila | 1 |
|  | Eschericha coli | 1 |
|  | Nocardia asteroides | 1 |
| Mycobacteria (n=3) | Mycobacterium avium complex | 1 |
|  | Mycobacterium abscessus | 1 |
|  | Mycobacterium fortuitum | 1 |
| Fungal (n=8) | Aspergillus fumigatus | 5 |
|  | Pneumocystis jirovecii | 1 |
|  | Penicillium spp | 1 |
|  | Candida glabrata | 1 |
| Viral (n=14) | Parainfluenza virus | 5 |
|  | Rhinovirus | 4 |
|  | Human metapneumovirus | 2 |
|  | Respiratory syncytial virus | 2 |
|  | Coronavirus OC43 | 1 |
|  | Cytomegalovirus | 1 |
| No pathogen identified (n=49) |  |  |

*Includes all results from microbiological studies; some patients had more than one organism that was implicated to cause pneumonia, while others had pneumonia syndrome without an isolated respiratory pathogen
